# The attitudes and practices of United Arab Emirates consumers towards food waste: A nationwide cross-sectional study

**DOI:** 10.12688/f1000research.135678.1

**Published:** 2023-07-31

**Authors:** Lynne Kennedy, Samir Safi, Tareq. M. Osaili, Ala Al Rajabi, Ayesha Alblooshi, Dima Al Jawarneh, Ahmed Al Kaabi, Fakhra Al Rubaei, Maitha Albreiki, Maryam Alfadli, Aseilah Alhefeiti, MoezAlIslam Ezzat Faris, Kholoud Allaham, Sameeha Junaidi, Moien AB Khan

**Affiliations:** 1 Public Health and Nutrition College of Natural and Health Sciences, Zayed University, Dubai, Dubai, United Arab Emirates; 2 Department of Analytics in the Digital Era, United Arab Emirates University, Al Ain, Abu Dhabi, 15551, United Arab Emirates; 3 Department of Clinical Nutrition and Dietetics, College of Health Sciences, University of Sharjah, Sharjah, Sharjah, 27272, United Arab Emirates; 4 Department of Human Nutrition, College of Health Sciences, Qatar university, Doha, P.O. Box 2713, Qatar; 5 Department of Health Sciences, College of Natural and Health Sciences, Zayed University, Abu Dhabi, P.O. Box 144534, United Arab Emirates; 6 Nutritional Studies Research Group, Department of Family Medicine, College of Medicine and Health Sciences, United Arab Emirates University, Al Ain, Abu Dhabi, 15551, United Arab Emirates; 7 Institute of Public Health, United Arab Emirates University, Al Ain, Abu Dhabi, 15551, United Arab Emirates; 8 Department of Neurology, Rashid Hospital, Dubai, Dubai, 4545, United Arab Emirates; 9 Department of Public Health, RAK medical and Health Sciences, Ras Al Khaimah, United Arab Emirates

**Keywords:** Household; Consumer, Food Waste; United Arab Emirates; Sustainable Development Goals; Environment; Culture; Incentivising Behaviour

## Abstract

**Background:** Reducing global food waste is an international environmental, health, and sus-tainability priority. Although significant reductions have been achieved across the food chain, progress by UAE households and consumers remain inadequate. This study seeks to understand the association between consumer attitudes, knowledge, and awareness relating to food waste practice of residents living in the UAE. to help inform policy and action for addressing this national priority. **Methods:** A cross-sectional study was conducted using a validated semi-structured online questionnaire through stratified sampling (n =1052). The Spearman correlation coefficient was performed to determine the correlations. Two independent regression analysis were used to determine the association between food waste practice with: 1) knowledge and awareness and attitude subdomains, and 2) sociodemographic characteristics. Respondents (n=1072) largely reflect the socio-demographic characteristics and population distribution across the seven Emirates. **Results:** As expected, a significant and negative correlation was found between food waste practice knowledge and awareness and overall attitude. The regression models showed reduced food waste practice was associated with better knowledge, personal attitude, financial attitude (first model), older age and fewer adults in the household (second model). We found a significant and negative association of personal attitude (a commitment, intention), financial attitude (cost-saving motivation), and (existing) knowledge of Food waste (FW) with practice of food waste, indicating that better knowledge about FW, personal attitude or financial attitude was associated with reduction in undesirable food waste practice. While awareness and emotional attitude (moral concerns) were positively and significantly associated with food waste practice (undesirable behaviour). **Conclusions:** Food waste poses significant challenges in the UAE, and addressing it requires a comprehensive understanding of the multifaceted factors influencing consumer behavior. By promoting knowledge, fostering positive attitudes, and considering socio-cultural factors, policymakers can develop effective strategies to reduce food waste in households and contribute to sustainable development goals.

## 1. Introduction

A precise measure of global food waste (FW) is difficult to obtain however according to a report by UNEP and WRAP, an estimated 931 million tonnes of food were wasted in 2019. ^1^ An amount arguably sufficient to feed the world’s hungry and malnourished twice over. ^2^ FW not only reduces the amount of food and drinking water available for human consumption but removes essential macro and micro-nutrients from the human food chain with severe consequences for human growth, development, and survival. FW therefore undermines the foundation of food and water security globally, highlighting the unethical distribution and consumption of finite planetary resources. FW is a major contributor to our global food-related carbon footprint ^3^^,^^4^; causing deforestation, desertification, and depletion of natural land and water resources locally. ^5^ FW contributes to greenhouse gas (GHG) emissions, and is therefore directly associated with climate change and global warming; as the largest waste component sent to landfill, FW decomposes anaerobically and produces methane, the greenhouse gas, which is reportedly a 25 times more potent pollutant to the ozone compared with carbon dioxide. ^3^ The economic losses associated with FW for farmers, landowners, producers, and retailers are also considerable, ^6^^,^^7^ reducing staple food supplies and foods for exports, a cost passed onto consumers. The environmental, economic, ethical, and social cost of FW to individuals, societies, and globally are therefore considerable. Furthermore, FW poses major challenges in terms of meeting global and multilateral commitments such as the 2014 Paris Climate Agreement and the United Nations (UN) Sustainability Development Goals (SDGs). ^8^ Unless urgent action is taken to prioritise the reduction of household FW, especially in specific regions of the world, including the UAE, then global objectives to halve food waste by 2030 (SDG 12.3; Responsible Consumption and Production (SDG 12), will be unachievable. Moreover, as this year’s host country for COP 28, all of the world’s attention will be focused on practices in this region.

The United Arab Emirates (UAE) in the Middle East and North African region (MENA), has a population of almost ten million and globally is reportedly a leading producer of food waste (FW). Regionally the UAE is the third largest producer of FW, behind Saudi Arabia, which is five times larger than the UAE, and the largest and most populated country in the GCC, and then Egypt. ^9^ With an estimated annual 95 kg food waste/per capita, the average UAE household generates some 923,675 tonnes of FW annually. ^10^ The two Emirates of Abu Dhabi and Dubai have a combined food waste of approximately 12.84 million tonnes annually, ^11^^,^^12^ equivalent to 1/130th of the previously estimated total annual global food waste (1.3 billion tonnes/p.a.). ^3^ In response, the UAE government has declared urgent action to reduce its carbon footprint, setting ambitious targets towards achieving the SDGs by the year 2030. ^13^ Policymakers have actioned its commitment to tackling food waste across the food chain ( https://www.foodwastepledge.ae/), focusing on the consumer end stage where the greatest losses are known to occur. Considerable progress has been made within the hospitality sector in the UAE, with highly innovative and successful programs and initiatives reported (see below), however the hospitality sector only represents one of the two key elements of FW at the consumer end stage. Therefore, achieving the UAE’s target of reducing food waste by 50% by 2030 to meet the country’s food security strategy, and UN sustainable development goals, policymakers are now keen on targeting household food waste, where the amount of food waste is both significant and largely avoidable. This will require a shift in consumer behaviour, based on a greater understanding of the factors influencing food waste by consumers and households, including more reliable estimates of FW by households and individual consumers.

### 1.1 Literature review

The terms *food loss waste* (FLW), food loss (FL) and food waste (FW) are all used interchangeably in the literature, however *Food Loss* is commonly used to refer to upstream losses, the reduced amount of edible food, quantity or quality, *occurring during initial production, postharvest and processing stages of the food chain;* whilst the term *Food Waste*, refers to any raw or cooked quality foods, including inedible items (i.e., skin, seeds, etc.) that is produced for human consumption, and then *discarded at the procurement and consumption stages.* ^11^^,^^14^ For the present paper we focus on FW, however it is important to consider how FW relates to the wider elements of this concept. Lipinski *et al.* (2013), characterised the term FLW as occurring across the entire food chain, with amounts lost or wasted varying both by country and region, or by commodity (key food groups, bread, starchy cereals, fruits, vegetables, etc.). More importantly, whether FLW is classified further i.e., as FW or FL appears to be influenced by several factors, namely the characteristics of a population, a country’s stage of economic development (developed, developing) or measure of GDP, climate, and political stability. Low-income or developing countries for example are more likely to incur the greatest losses upstream, i.e. Food Loss (FW), at pre or post-harvest/pre-processing stages i.e. Food Loss, primarily due ^9^ to issues relating to inhospitable climates, drought, water scarcity, inadequate storage and distribution infrastructures, war and civil unrest. In contrast, industrialised or developed countries, including countries in the MENA region, such as the UAE, typically experience the greatest food wastage at post-production – i.e. downstream loss, in the food retail and hospitality sector and at the household consumption stage. Almost a third (28%) of the FLW produced by developed countries, including the UAE, occurs at the consumption stage, and consists mostly as household waste, compared to just 7% consumer stage waste for developing countries. ^9^ A level associated with food insecurity, a lack of available foods, and in response more frugal food management strategies. Many developed countries, particularly those located in the Northern hemisphere, have successfully implemented policies to reduce waste in the retail and hospitality sectors, and with some success in changing consumer behaviour, resulting in FWL at the consumption stage, similar to developing countries.

This is not the case however for some countries in the southern hemisphere, which have experienced increased development and affluence. Considerable progress has been made by the UAE government in addressing FW within the food retail and hospitality sectors. Upstream action directed at addressing FW, i.e. at the consumption stage of the food chain, including household food waste (HFW), is however an under-developed area. ^15^ The UAE government has invested substantial funds to support multi-sectoral partnerships to create innovative and sustainable food waste solutions, with additional investment promised. ^16^ One example, Winnow, ^17^ has helped save an estimated three million meals associated with FW. Significant success with food repurposing efforts is also evident. ^18^ Whilst this action is commendable, the savings are insignificant compared with the overall costs associated with downstream losses, i.e., FW, in the UAE, since the value-added lost to waste is the highest at the consumption stage; moreover, when approximately 90% of all food consumed in the UAE is imported the value-added losses are even more considerable and therefore critical to address. Thus, calls have been made for government and industry to partially shift some of the current emphasis on targeting hospitality and retail, to focus on the neglected component of FW arising at the household or consumer end of the food chain. Such calls have been echoed by other countries with similar FW profiles, i.e. countries that have experienced rapid economic development, such as Saudi Arabia and Qatar, who rely on importing the majority of its food. In order to effectively address FW in the UAE, a better understanding of consumers FW behaviour and the various factors influencing this is a fundamental requirement. As part of the present study, we have reviewed the FW literature, with specific reference to studies undertaken in countries similar to the UAE, in the MENA or Arab speaking region.

Regardless of social context, FW at the household level (HFW) occurs at three stages, (a) after purchase but before preparation, (b) between preparation and serving, or (c) after serving (as leftovers). ^19^^,^^20^ In each of these stages a variety of factors have been identified as influencing food waste and consumer’s behaviour, which in turn is directly influenced by a range of socio-demographic and environmental factors. ^21^ All human behaviour including HFW is socially and culturally nuanced, so research aimed at understanding HFW must be socially situated, in order to provide insight into the sociocultural practices in that specific context. Moreover, the UAE is socially and culturally unique with an extremely diverse population; depending on the specific Emirate, 80-90% of the population are expatriates, originating primarily from India, Pakistan, Philippines, USA, Canada, and Western Europe, respectively. Some of these cultures, including Emirate households, typically cohabit with non-family members, as intergenerational families, whilst others live as smaller and homogenous units. Household composition affects how food is accessed, procured, stored, cooked, consumed, and wasted. In order to understand how the sociocultural context and demographic factors influence HFW in the Arab region, and the UAE specifically, we created a conceptual framework from HFW studies conducted in The Near East and North Africa (NENA) and MENA regions. There was a distinct paucity of studies pre-2010, before the establishment of the UN SDGs and calls ^22^ for more research were made. In the past decade the number of published studies has increased, including several systematic reviews. ^7^^,^^23^ The majority of these studies are undertaken in Egypt or Saudi Arabia with only limited research undertaken by the smaller Arab countries. This particular body of literature also examines a wide range of factors relating to HFW: levels of HFW; consumer or HFW practice (i.e. behaviours relating to or resulting in HFW) and the knowledge, attitudes, intentions, motivations, relating to HFW practice; household food management practices; and strategies associated with reducing HFW. More research is needed to increase understanding about consumer behaviour as it relates to HFW, including individual’s attitudes, beliefs, and motivations towards HFW behaviour, but especially studies specific to the UAE’s heterogenous population and socio-cultural context.

It is widely accepted that socio-cultural, economic, and socio-demographic factors are directly linked with HFW. Household income, size and composition are all acknowledged as key determinants of HFW ^24^ as age, level of education, and income significantly influence intention to reduce food waste. Households in rural areas are likely to consume food prepared at home, compared with those living in urban areas who favour convenience, processed i.e. value-added foods and imported foods; being employed outside the home, or self-employed, is positively correlated with food waste; eating out and purchasing food on special promotion have been linked with regular and higher levels of FW. ^25^

Understanding consumer behaviour is also key to developing a better understanding of HFW and how to prevent or reduce this. Many attempts to understand consumer behaviour, in any area, have utilised behavioural theories as a conceptual framework, with varying success. Several Arab-based studies of HFW have used Ajzen’s (1991) Theory of Planned Behaviour (TPB). The basic theory of TPB is an explanatory or predictive model that hypothesises consumer behaviour is directly influenced by an individual’s ‘attitudes towards a particular behaviour’ (positive or negative evaluations of self-performance relating to a behaviour; the influence of ‘subjective norm’ or an individual’s response towards social pressure to conform, or significant others, to conform to particular behaviour; ‘perceived behavioural control’ or a belief in one’s ability to perform the (desired) behaviour (i.e. self-efficacy) and perceived behavioural control or the perceived ease of difficulty in behaving a specific way. ^26^ For example, an individual’s motivation or intention to reduce FW might be determined by their predisposition towards waste control (subjective norm) i.e. whether society or significant others value FW avoidance behaviour. As one study in Tehran ^27^ reported, the TPB was helpful in directing attention on factors influencing practice and they found that good food management skills (food procurement, food storage and handling, etc.,), may be a strong predictor of HFW behaviour; this however might be moderated by the trade-off between knowing what is considered socially acceptable (social norms) and confidence in their ability to adopt and carry out good management practices (self-efficacy). Whilst the TPB has been successfully used to help explain consumer behaviour in the field of FW, some researchers have found that the model only partially explains variations in participants behaviours. For example, Karim-Ghani *et al.*, 2013, explained 13.7% of variation in consumers’ intention to adopt waste separation behaviour to avoid FW. Whilst Chalek *et al.* 2016, cited in Aktas *et al.* 2018, used TPB to help determine regional differences in FWL, estimating almost 65% of the variation between country-level food waste could be determined by gross national income. ^28^^–^^30^ Researchers have argued that consumer management skills (CMS) and specifically food management strategies, directly influence FW behaviour, for example, consumers who make shopping lists are less likely to over-purchase ^31^ whilst consumers who regularly over-purchase and buy more food than needed, are enticed by special offers and promotions and potential economies of scale from buying in bulk, are more likely to waste food, due to poor ‘food control management’ practices (FCM); this however may be moderated by certain attitudes for example anxiety relating to the cost of FW and feeling guilty as a result of over-buying, is negatively associated with household FW loss. ^32^ With the latter found to be important in studies conducted in the Arab region, as mentioned below. Consumers who tend to adopt less healthy dietary patterns have been shown to positively influence FW, yet higher economic status may increase the relative frequency or occurrence of household food waste linked with over-purchasing behavior and food abundance due to increased affordability of food for high-income households. ^33^ Since our initial review of the literature suggested a contradiction around affluence and financial attitudes, including food management behaviour, associated with cultural traditions around hospitality, we considered utilising TPB as a general framework, whilst also including focus on the wider socio-cultural literature on FW behaviour, particularly relevant in studies undertaken in the Arab region, and not restricting our study to this framework. The development of the present study was therefore influenced by the wider literature on FW practices and behaviour, values, beliefs, and attitudes across MENA.

Diaz-Ruiz *et al.* (2018), identify six predictors of HFW: environmental awareness; materialism (consumerism); purchasing behaviour; dietary choice; attitudes towards waste recycling; and waste prevention. ^34^ Abdelradi (2018) adopts this framework, extending this to include knowledge of food waste as a problem, personality traits and religion; this aligns with Elshaer *et al.* (2021) who posited that religion is a key predictor of adverse HFW in Saudi and therefore the wider Muslim region. ^35^ One explanation for religiosity as a predictor of adverse FW behaviour is the importance of frugality, morality and altruism in religious codes, ^36^ which reportedly contradicts with respondents aspiration to be perceived as a ‘good provider’, as a precursor to increased food waste. The cultural importance of Arab traditions rooted in food, hospitality and expressions of generosity may also accentuate the influence of religiosity as a determinant of HFW in the UAE and especially in countries where the population is predominantly Muslim; whereby people who have a positive and moral attitude towards the environment and the importance of food waste have a greater intention to reduce food waste, yet this contradicts with pressures associated with cultural traditions (hospitality). This aligns with Hedari *et al.* (2020), who suggested that this influence is developed through the effect on subjective norm and attitude, and waste-avoidance or prevention behavior may be a stronger predictor of intention to reduce household food waste than previously thought; therefore, interventions to reduce consumer food waste should concentrate on establishing waste avoidance attitudes. ^37^ This concurs with a study conducted in the U.S., ^38^ whereby consumers who are aware of the consequences of food waste are also more receptive towards food waste reduction. Suggesting that considering the consequences of food waste, i.e. economic cost, needless hunger, and climate change, may encourage consumers to act sustainably and avoid waste. A sense of community has also been found to encourage food waste reduction, repurposing and recycling behavior. However, a study of youth in Pakistan suggests people who perceive they are excessively busy are significantly less likely to engage in the same food waste avoidance behaviors. ^38^ Although knowledge, attitudes and practice influence the participants’ decisions in determining what types of food are appropriate for them and their families to consume and which foods should be discarded. Despite this understanding of food labels with respect to food shelf-life is limited amongst consumers, thus, more food than necessary is discarded. ^39^ Having a strong sense of social responsibility, including particular religious values or environmental concerns, is also reported with conserving food and reducing food waste behaviours. ^40^ Food-related practices (procurement, cooking, storage and consumption) of a household vary according to cultural values, beliefs and socio-economic status (SES) i.e., educational attainment, occupational and income status. ^41^ Research also suggests that food waste may be higher in households where the woman is employed outside of the home ^42^ and whilst women may traditionally stay at home, in the UAE, where the majority are expatriates, a high proportion of women are in employment.

The rapid societal and economic transition experienced by oil-producing Arab states, from traditional nomadic to wealthy industrialized societies, may compound consumer food waste behavior in countries such as the UAE. Baig *et al.* (2019), reported how HFW in Saudi Arabia, the leading producer of HFW regionally, is positively associated with economic affluence. In addition to cultural traditions (i.e. the need to express hospitality), widespread lack of awareness about issues relating to FW and the absence of policies targeting consumers to reduce FW, is believed to compound food waste behavior. ^43^ As a study undertaken in KSA ^44^ explains, how surplus food is classified as ‘unclean’ or ‘bayt’ by its people is an important determinant and predictor of HFW. This is especially relevant for families who eat from individual rather than traditional practice of communal plates. The sensory properties of food are key when deciding whether to use ‘bayt’ food (cooked food that is kept for at least one night). Leftovers are sometimes deemed unwanted because of the desire to avoid eating the same food on consecutive days. Although ‘bayt’ food is perceived as less desirable, it is considered edible and so it is haram (forbidden) to waste it. This raises a dilemma, in choosing enjoyment by serving fresh food vs. performing duties as Muslims by reusing edible leftover, or ‘bayt’, food. To escape the sin of waste, some interviewees reported passing unwanted food to their house staff or forcing themselves to eat it. During Ramadan meat-less surplus food becomes bayt and is unwanted because it is a ‘light’ dish. The authors also explain that rapid economic development, resulting in an affluent population has directly encouraged surplus generation and the attractiveness of leftovers. Where a preference for abundance and the multiplicity of choices, but also having the option not to consume leftovers, are related to this rapid rise in affluence. Social norms, as our data suggests, are more important than economic status in the rejection of surplus food. In Saudi, eating flavorless food was once common, today, however, it is no longer the norm to eat and serve such food – even amongst less affluent interviewees. Reliance on restaurants while eating outside the home during Ramadan also results in greater amounts of home-cooked foods being rejected. Whereas the fear and embarrassment of being criticized or bullied can deter some people from repurposing household food and taking leftovers to consume outside of the family home. ^44^

As the current literature suggests many factors are involved in determining consumer food waste and encouraging food waste avoidance behaviour. These factors are also socially and culturally situated and therefore require context specific research. Efforts to reduce household food waste therefore require research that helps illuminate the determinants of FW behaviours, including knowledge and attitudes towards household FW, amongst consumers living in the UAE. The present study will address this by contributing to the understanding of consumer knowledge, attitudes and behaviours relating to FW in the region. Based on our review of the current FW literature in this region, we can assume that FW is positively associated with certain socio-demographic characteristics, such as age, social status and household income, including anxiety relating to food insecurity by low-income households; household composition, ethnicity; religious beliefs; cultural values; knowledge and attitudes towards food waste and food waste avoidance. FW behaviour is also likely to be directly influenced by consideration of social norms related to FW; consumers who adopt highly processed, less traditional middle eastern or Mediterranean dietary patterns are likely to be positively influenced to FW compared with those who adopt traditional dietary patterns and traditions, including acceptance for repurposing foods i.e. ‘bayt’; as is with lack of knowledge and awareness of food and nutrition labelling or the nutrition content of foods; and poor household food-management skills. As stated, the purpose of the present study is to provide much needed baseline data collected in the UAE on consumers attitudes, opinions, knowledge and practice (behaviours), relating to household food waste and is guided by the above conceptual framework.

## 2. Methods

### 2.1 Study design and sample

A cross-sectional study was undertaken to collect data on household food waste knowledge, attitudes and practices (KAP) from people within the UAE. For inclusion, participants needed to be UAE residents aged 18 or above. The minimum sample size was calculated to be 637 with a population of 9.89 million, with a margin of error of 5%, 95% confidence interval (95% CI) and response rate of 40%. ^45^

### 2.2 Sampling and recruitment strategy

To obtain a representative sample of the population in the United Arab Emirates, a stratified sampling technique was employed. The population of the emirates was divided into three geographical areas: Abu Dhabi, Dubai, and the Northern Emirates. Each geographical area was considered a stratum. Participants were randomly selected from each geographical area in proportion to their population. To increase the representativeness of the sample with respect to households in the UAE, survey responses were collected from the UAE resident population in the three main zones: Abu Dhabi, Dubai and Northern Emirates. The survey was created using google forms. The survey was distributed via email and social media platforms to all participants. The survey was accessible via an anonymous link between 14 ^th^ December 2021 and 8 ^th^ February 2022. At the end of the study period, the survey was automatically disabled. Collaborators from each emirate collected the data to ensure that it was representative of the population. A question in the survey response inquired about the participants’ current residence.

### 2.3 Ethical approval

The Social Sciences Research Ethics Committee (REC) from the United Arab Emirates University (UAE-U) approved the study (reference number is ERS_2021_8380). The study was conducted in accordance with Helsinki Declaration guidelines. ^46^ Informed consent was obtained from all participants involved in the study prior to commencing the online survey. They were provided with clear and detailed information about the purpose of the study, the procedures involved, and how their data would be used and published. Participants were explicitly informed that their identities would remain confidential and would not be disclosed in any publications or presentations resulting from the study. The focus would solely be on the aggregated data and group-level findings, ensuring anonymity and privacy for each individual participant.

### 2.4 Data collection instrument

The design of the semi structured survey was informed by relevant existing validated questionnaires from the literature. ^7^^,^^35^^,^^47^^–^^51^ Four experts from the field were requested to rate the questions on a scale of 1 to 10 for clarity, validity (ability to meet study objectives) and reliability. Any question which scored below seven on any aspect was removed. Feedback from the experts was addressed to strengthen the survey design. Reliability was tested using Cronbach Alpha. A reliable score of 0.80 was observed. The questionnaire was bilingual, English and Arabic; with forward and backward translation from English to Arabic undertaken by bi-linguists as per the methodology. ^52^ The final survey, with Arabic translations, was piloted on 30 candidates for comprehensiveness and clarity. No major issues were received. The structure of the survey included a clear statement of the details of the study, necessary for informed consent, which was obtained from participants prior to starting the questionnaire. Participants were advised that they could exit the study by quitting the survey at any time, without consequence.

The questionnaire collected data on sociodemographic characteristics, and food waste knowledge, attitudes, and practices (KAP). For KAP-related questions, participants were asked to score their responses based on a five-point ordinal Likert scale which was later computed (mean) to estimate knowledge and awareness, attitude, and practice scores **(Supplementary Table 1)**.

*2.4.1 Knowledge and awareness*

The knowledge subdomain had two items while the awareness subdomain had a single item **(Supplementary Table 1)**. Participants knowledge, and participants knowledge and awareness, were assessed by calculating the mean score of the two knowledge items and the three items of the knowledge and awareness subdomains, respectively. Higher scores reflected better knowledge and awareness regarding food waste (i.e. desirable behavior).

*2.4.2 Attitude*

Validated questions related to personal, emotional, behavioral, and financial subdomains of the attitude towards food waste were used and contained five, three, two, and four items, respectively **(Supplementary Table 1)**. ^35^^,^^47^^,^^48^^,^^50^ Participants ‘personal, emotional, behavioral, and financial attitudes were assessed by calculating the mean score of the items included in their respective subdomains. The overall attitude was assessed by calculating the mean score of all fourteen items included in all four attitude subdomains. Higher scores reflected better attitude regarding food waste (i.e., desirable).

*2.4.3 Practice*

The practice domain had fifteen items **(Supplementary Table 1)**, and their mean score was used to assess participants’ practice of food waste. Higher scores reflected worse practice of food waste (i.e., undesirable).

### 2.5 Data analysis

Data was analyzed using the statistical software Statistical Package for the Social Sciences (SPSS) (version 28). ^53^ The mean and standard deviation (SD) were used to summarize continuous data. The mean percentage was calculated as a percentage of the maximum possible score. Categorical data were presented using frequencies and percentages. Spearman rank correlation coefficients were utilised to examine correlations between practice and each of knowledge and awareness, total attitude, and their subdomains. Furthermore, multiple regression model was used to examine the association of all independent variables (knowledge, awareness, personal attitude, behavioral attitude, emotional attitude, and financial attitude) with practice. In a separate analysis, multiple regression model was used to examine the association of sociodemographic characteristics (gender, age, number of adults in household, marital status, area of residence, number of children, educational level, and household income) with practice. The 95% confidence intervals (95% CIs) were used to determine the strength and direction of the associations. Type I error was fixed at 5% for all tests.

## 3. Results

### 3.1 Sociodemographic characteristics and household composition

A total of 1052 participants were included in the study. A total of 70.9% of participants were from Abu Dhabi, 17% from Dubai, and 12% from Northern Emirates (Sharjah, Ajman, Umm Al Quwain, Ras Al Khaimah and Fujairah). The distribution of the survey responses matches the distribution of the population and the area of the UAE **(Supplementary Figure 1)**. The majority of participants were female (76%) aged between 18 and 30 years (58.2%) **(Supplementary Table 2).** The lowest response rates 1.3%, 6.6%, and 6.6% were received from ages 61-70, 51-60 years, and 41-50 years respectively. The most common household compositions reported by participants were homes of seven or more adults (25%) and two adult households (19.8%). Additionally, six (12.1%), five (13.6%), four (15.5%), three (10.1%) and one adult (4%) household occupancy were reported. Over half (54.8%) of the participants were single compared with 42.6% married. Whilst only 0.3% and 2.4%, were reported to be widowed or divorced, respectively. The majority of participants lived in the city (87.4%), while 7.3%, and 5.3% of participants living in towns and villages, respectively **(Supplementary Table 2).**

Nearly a third (27.5%) of participants lived in households without children, while 56.1% lived in households with 1, 2 or 3 children (19.9%, 18.7%, and 17.5%, respectively). A total of 17% of the participants lived in households with more than three children (four, five, or six children, 9.5%, 3.7%, and 3.1%, respectively). The majority of participants were reportedly educated to degree level, with most having undergraduate degrees (65.8%), whilst almost a quarter of the sample (23.3%) reported having postgraduate degrees and 8.7% had a PhD or higher. A minority (0.6%) reported having attained less than secondary school education, 1.7% less than high school education. Over half of participants (55.7%) reported basic middle income, 13.4% marginal middle income, 24.6% upper middle income, 2.4% lower income, and 4.2% upper income **(Supplementary Table 2).** The full anonymised dataset can be found under *Underlying data.* ^66^

### 3.2 Knowledge and awareness, attitude, and practice

In **Supplementary Table 3**, we present the descriptive data and analysis of estimated self-reported food waste-related Knowledge and Awareness, Attitude, their respective subdomains, and Practice.

Among those surveyed, mean (95% CI) knowledge, awareness and knowledge and awareness combined were 83.00 (81.91- 84.10), 63.29 (61.92-64.65), and 76.43 (75.56-77.30), respectively. Mean (95% CI) personal, behavioural and emotional attitude “toward avoiding” food waste were all above 80% [84.32 (83.55-85.09), 87.60 (86.81- 88.40), and 83.95 (83.01-84.90), respectively], with mean (95% CI) financial attitude of 68.89 (68.26-69.53). Mean (95% CI) overall attitude was 80.56 (80.00-81.13). Practices of food waste had a mean (95% CI) of 19.92 (18.77-21.07).

### 3.3 Spearman’s Rank Correlations

In Table 1, we show the correlations between practice as a response variable and knowledge, awareness, knowledge and awareness, personal attitude, behavioural attitude, emotional attitude, financial attitude and overall attitude, as independent variables. The correlation coefficients indicate the strength and direction of the relationship between two variables.

**Table 1.** Correlations between practice and independent variables.

Domains	No adjustments		Partial correlations (adjusted for age)	
	Spearman's rho correlation coefficient (95% CI)	p-value	Spearman's rho correlation coefficient (95% CI)	p-value
Practice - Knowledge	-0.155 (-1.00, -0.104)	<0.001 **	-0.169 (-0.235, -0.1)	<0.001 **
Practice – Awareness	0.005 (-0.048, 1.00)	0.44	0.041(-0.016, 0.097)	0.18
**Practice - Knowledge and Awareness**	**-0.123 (-1.00, -0.072)**	**<0.001** **	**-0.12 (-0.185, -0.049)**	**<0.001** **
Practice - Personal Attitude	-0.303 (-1.00, -0.255)	<0.001 **	-0.284 (-0.342, -0.222)	<0.001 **
Practice - Behavioral Attitude	-0.237 (-1.00, -0.187)	<0.001 **	-0.198 (-0.261, -0.132)	<0.001 **
Practice - Emotional Attitude	-.051 (-1.00, 0.002)	0.051	-0.075 (-0.135, -0.007)	0.015 *
Practice - Financial Attitude	-0.165 (-1.00, -0.114)	<0.001 **	-0.163 (-0.217, -0.108)	<0.001 **
**Practice – Overall Attitude**	**-0.276 (-1.00, -0.228)**	**<0.001** **	**-0.269 (-0.326, -0.210)**	**<0.001** **

** Correlation is significant at the 0.01 level (1-tailed).

* Correlation is significant at the 0.05 level (1-tailed).

The undesirable food waste practice was negatively and significantly correlated with (Spearman’s rho correlation coefficient, p-value) knowledge (-0.155, <0.001), knowledge and awareness (-0.123, <0.001), personal attitude (-0.303, <0.001), behavioural attitude (-0.237, <0.001), financial attitude (-0.165, <0.001) and overall attitude (-0.276, <0.001). The significance of all the correlations continued even after controlling for the effect of age, as shown in Table 1. This indicates that age did not have a significant impact on the observed associations between the variables. These results indicate that an increase in each of these variables (knowledge, knowledge and awareness, personal attitude, behavioural attitude, financial attitude and overall attitude) is correlated with a reduction in food waste practice. The correlation between practice and emotional attitude was not significant (-.051, 0.051), however; it reached significance after adjusting for age (-0.075, 0.015). Overall, the results suggests that better attitude, knowledge and awareness are correlated with reduction in food waste practice.

### 3.4 Correlation between Practice and Knowledge, Awareness, Attitudes

*3.4.1 Multiple Regression Models*

We ran a multiple regression model to further clarify the associations of all independent variables (knowledge, awareness, personal attitude, behavioural attitude, emotional attitude, and financial attitude) with practice as the dependent variable ( Table 2). Analysis of variance (ANOVA) for the multiple regression model indicated that at least one of the independent variables (the six subdomains) is significantly associated with the predictor variable (practice domain) (F=21.563, p-value <0.001; **Supplementary Table 4**).

**Table 2.** Multiple regression model results on the association between practice as the dependent variable and all six subdomains as independent variables.

Subdomain	Regression coefficient	95% CI	p-value
Knowledge	-0.077	-0.128, -0.027	0.003 **
Awareness	0.046	0.007, 0.085	0.021 *
Personal Attitude	-0.282	-0.374, -0.189	<0.001 **
Emotional Attitude	0.074	0.008, 0.141	0.029 *
Behavioural Attitude	-0.067	-0.161, 0.027	0.163
Financial Attitude	-0.150	-0.237, -0.063	0.001 **

** Statistically significant at 0.01 level.

* Statistically significant at 0.05 level.

Results of the multiple regression models showed that (regression coefficient, p-value) knowledge (-0.077, 0.003), personal attitude (-0.282, <0.001), and financial attitude (-0.150, 0.001) were significantly and negatively associated with food waste practice, indicating that better knowledge, personal attitude, or financial attitude was associated with reduction in the undesirable food waste practice. While awareness (0.046, 0.021) and emotional attitude (0.074, 0.029) had positive significant association with food waste practice.

We ran a seperate multiple regression model to assess the associations of sociodemographic variables (gender, age, number of adults in household, marital atatus, area of residence, number of children, educational level, household income) with practice as the dependent variable ( Table 3). Analysis of variance (ANOVA) for the multiple regression model indicated that at least one of the independent variables (sociodemographic variables) is significantly associated with the predictor variable (practice domain) (F=5.983, p-value <0.001; **Supplementary Table 5**).

**Table 3.** Multiple regression model results on the association between practice as the dependent variable and sociodemographic variables as independent variables.

Sociodemographic	Regression coefficient	95% CI	p-value
**Gender**			
Male	Ref		
Female	-0.013	-0.124, 0.099	0.824
**Age (years)**			
51 and above	Ref		
18-30	0.211	0.004, 0.418	0.046 *
31-50	0.062	-0.125, 0.249	0.516
**Number of adults in household**			
7 or more	Ref		
1-3	-0.247	-0.386, -0.108	0.001 **
4-6	-0.204	-0.326, -0.082	0.001 **
**Marital status**			
Single, divorce, & widowed	Ref		
Married	-0.040	-0.169, 0.088	0.536
**Area of residence**			
Town and village	Ref		
City	-0.023	-0.163, 0.118	0.750
**Number of children:** continuous	0.023	-0.007, 0.054	0.128
**Educational level**			
Undergraduate & less	Ref		
Postgraduate & higher	-0.101	-0.210, 0.007	0.067
**Household income**			
Upper middle & upper class	Ref		
Lower, marginal middle & basic middle	-0.047	-0.150, 0.057	0.377

** Statistically significant at 0.01 level.

* Statistically significant at 0.05 level.

Results from the second multiple regression model (regression coefficient, *p*-value) indicated that being 18-30 years old is significantly and positively associated with higher food waste practice compared to those 51 years old and above (0.211, 0.046). In addition, having 1-3 (-0.247, 0.001) or 4-6 (-0.204, 0.001) adults in the household was associated with less food waste practice compared to households with 7 or more adults. No other associations reached statistical significance.

## 4. Discussion

Using a nationwide cross-sectional survey of adults residing in the UAE, we recruited 1072 participants from the seven Emirates of the UAE. Approximately two thirds of the survey sample were collected from the Abu Dhabi Emirates, which represents the majority of the UAE’s land area and population distribution, while the rest came from other Emirates **(Supplementary Figure 1)**. Our sample is skewed towards the most populated Emirates of Abu Dhabi and Dubai, (70.9%; 17% respectively), with only 12% of participants recruited from the five much smaller Northern Emirates, however this is representative of the geographic distribution. The majority of participants were single (55%), female (76%), aged 21-30 years (40%), living in households of multiple occupancy, with 7 or more adults, and living in the city (85%). A third of our sample lived in households without children, whilst most (56%) were small households, with 1-3 children; the educational attainment of respondents was above average, with two-thirds of participants educated to degree level, a further 23% and 8% achieving postgraduate or doctorate degrees, which is higher than expected in terms of the general population and suggests recruitment bias towards highly educated/skilled ex-pat workforce. Household income was also skewed towards middle income (55.7%) and upper middle income (24.6%) compared with just 2.4% for lower-income households. This may reflect above average ex-pat population in these two Emirates, and since we know that the UAE population is extremely diverse and heterogenous, is representative of the wider UAE population. Nonetheless, future surveys should seek to recruit a larger proportion of Arab and Emirati Nationals, living in Abu Dhabi and Dubai. In the absence of data on consumers and food waste within the UAE, our findings are still useful in highlighting potential trends and suggesting areas for further research.

The purpose of the present study is to explore the level of participants knowledge and awareness, attitudes, and practice of food waste (FW); and factors associated with FW. We found a significant and negative association of personal attitude (a commitment, intention), financial attitude (cost-saving motivation), and (existing) knowledge of FW, with practice of food waste, indicating that better knowledge about FW, personal attitude or financial attitude was associated with reduction in the undesirable food waste practice. While awareness (0.046, 0.021) and emotional attitude (0.074, 0.029) had a positive and significant association with food waste practice, as the undesirable behaviour.

Our findings align with the wider literature on FW behavior, knowledge and awareness whereby the majority of respondents are aware that FW is an important issue. The mean for awareness (63.3%), as an important societal issue, was relatively high and also the mean knowledge (76%) about FW. Despite this however, our study did not find a significant association between awareness and FW avoidance. As one recent systematic review reported, studies undertaken in this region suggest that knowing FW is problematic, and should be reduced, is not necessarily the most important predictor of behavioral intention; the authors suggest that practice may be more influenced by negative attitudes towards FW by other family members; lack of food management skills; lack of storage facilities; inconvenience of storing leftover foods, such as large (bulky) packaging of food items, combined with limited storage space; lack of knowledge of food safety, spoilage and food-poisoning risks, as practical factors hindering consumer intention to reduce food waste. ^54^ Additionally, in contrast to some researchers, we observed that concerns relating to guilt associated with food waste practice as measured by the domain emotional attitude, was not a significant motivator for behavior avoiding food waste in our sample. Nonetheless, personal attitude (domains relating to environmental concerns of food waste) was a significant motivator of practice, which concurs with. ^55^ Given the current increased media and government attention towards the importance of reducing food waste and SDG 12 within the UAE, and as hosts of the forthcoming COP 28 meeting in 2023, we might reasonably expect to see a positive association with practice (i.e., reduced food waste). Further research is recommended to better understand how public attitudes towards environmental concerns may provide effective leverage, or not, in shifting food waste practice in the UAE context. Additionally, if awareness is not significantly associated with food waste avoidance, then identifying the salient elements of environmental or personal attitudes, associated with FW avoidance practice, may offer promising alternatives.

As our data suggests, practice - food waste was significantly and positively associated with younger age (18-30 years) and household composition (households of multiple occupation, with more than 7 adults). The reference categories in Table 3 are used as a comparison point to interpret the coefficients for the other categories. For example, the coefficient for Female is -0.013, which means that, on average, females practice 0.013 times less than males, after controlling for the other demographic variables, and the coefficient indicates a small, non-significant difference between the genders in terms of practice. Similarly, the coefficients for the age categories (Age: 18-30 and Age: 31-50) are compared to the reference category of Age: 51 and older. The coefficients for Age: 18-30 and 31-50 are 0.211 and 0.062, respectively, indicating that, on average, individuals aged 18-30 and 31-50 practice 0.211 and 0.062 times more than individuals aged 51 and older, after controlling for the other demographic variables. Similarly, the coefficient for number of adults in house 1-3 and 4-6 are -0.247 and -0.204, respectively, indicating that, on average, households with 1-3 and 4-6 adults’ practice 0.247 and 0.204 times less than households with 7 or more adults, after controlling for the other demographic variables. Thus, efforts targeting younger generations in the UAE, and smaller households are worth considering. Since many of the cultures represented in the UAE population are traditionally patriarchal, targeting men and involving elders as social influencers, could also be useful for designing effective public campaigns and community-based intervention strategies. According to the literature FW behaviour is significantly associated with good household food management skills, food service/provision knowledge and skills, effective procurement (shopping) and cooking skills, in addition to knowledge, and attitudes. ^42^ Raising awareness and developing skills associated with avoiding FW could also target couples and households, rather than just women as the main homemakers.

A better understanding of consumers’ shopping behaviours is important for addressing the issue of food waste. We found that financial attitudes were negatively associated with practice (food waste avoidance). In the UAE, as in other developed countries, increased accessibility, abundance and variety of foods available to consumers encourages people to purchase more food than they need. ^11^^,^^56^ The literature suggests that overbuying of food is directly influenced by retailing offers, e.g., ‘buy one get one free’, multi-packs, however since the standard of living and average salaries, particularly amongst nationals who are supported financially by government, are amongst the highest globally, this may help explain why financial attitude was negatively associated with practice. Since the majority of people living in the UAE, especially ex-pats with higher degrees, as in this study, may not be overly price sensitive or price conscious, this may contribute negatively to over-purchasing and inadequate meal planning, leading to food waste. Failure to check expiry dates, or to estimate supplies needed, may also be a factor in the large volume of food waste, due to increased likelihood of food-spoilage ^24^ especially because of the short shelf life of many foods in the UAE climate. Some consumers purchase daily to ensure they are eating fresh food; others gather food for the whole week, which tends to be wasted, with more than half (50-75%) of households globally disposing of meal leftovers. ^11^

Understanding the problem of food waste from a consumer perspective may offer opportunities to reduce high rates of food waste. A recent study in the UAE showed that 96% of participants agreed that food waste is a problem ^11^ whilst many consumers regret wasting food. On the other hand, the literature suggests that many (60%) consumers do not believe that food waste is a serious issue since food is biodegradable. ^57^ As our data suggests, personal attitudes (concerns about the environmental issues) was significantly associated with FW avoidance practice. So further insight into the importance of this domain is recommended. In line with our observation, previous research suggests that individual attitude has a significant effect on food wastage behavior. Attitude towards food wastage has been observed to be influenced by the cost of food (i.e. financial attitude), based on prior experience of household food scarcity (food insecurity), societal norms, identity of self as a ‘good provider’, whether food is convenient or homemade, who was responsible for preparing the food, or the ease with which the food could be stored. ^58^^,^^59^ However, in contradiction to the “Theory of Planned Behavior”, we did not observe a clear and significant link between overall attitude and practice in our sample.

In order to address the problem of household FW in the UAE, it is evident that more research is needed to help understand consumer behavior and the factors influencing this, in the local context; Additionally, national policies and initiatives to influence consumer knowledge, attitudes and thus behavior are urgently required. Introducing information about the negative impact of food wastage in school curriculums may help educate children/youth and help encourage wider cultural shifts in FW behavior. Discussing the issue frequently on social media, newspapers or other multimedia channels would be a good reminder to the public. Government supported incentives/food banks/ competitions/cash rewards would also inspire the public to act. The awareness needs to be rigorous and planned in a manner that reducing food wastage becomes a permanent habit for at least some portion of the public. ^60^^,^^61^ Our research suggests more effort needs to be targeted at influencing consumer attitudes towards food waste; including supportive education around improving consumer knowledge about the shelf life, storage, preservation and packaging of different foods, to help reduce waste through spoilage; better food provisioning strategies, especially to avoid over-purchasing of foods in excess of normal and physiological need.

Future investments could be made in technology that automatically informs the user about expiration dates, items currently in the refrigerator/at home, and suggest recipes to utilise the foods currently available at home etc. Studying country case studies which have successfully tackled food wastage could prove to be of great help to develop future action plans for the country. ^58^^,^^62^ Building on the successful technological developments and mobile apps which help guide consumers to distribute excess food conveniently and safely to those in need are also promising, to help repurpose and distribute excess foods, thus helping to reduce household food waste.

To the best of our knowledge, this is the first study to look at consumer knowledge and practice relating to FW in the UAE and as such provides useful insight into this area and the factors influencing consumer behavior or intentions to reduce FW within this particular social and cultural context. A key limitation of the study relates to the challenges associated with sampling and recruitment. Since there is no formal postal service, even in major cities, the ability to survey households is restricted. This is not unique to the UAE and is an issue in similar countries in the MENA regions. Thus, researchers rely mainly on social media as a vehicle for disseminating surveys, or alternatively use personal contacts for convenience and snowball sampling. Governmental surveys and commercial survey companies exist, and could be approached to undertake a more representative national cross-sectional survey in the future. Nevertheless, with the intended outcome of the research and the types of factors used, such sampling with a minimal bias is considered acceptable. ^63^ This selection bias was reduced by using an optimal sample size. ^64^ A further limitation is the reliance on self-reported food waste behavior. Additionally, such self-reported food waste behavior might be subjected to social desirability bias by participants reporting what they consider morally acceptable instead of how much food is actually wasted. ^65^ Based on our aims, it is not feasible to conduct an observational study of food behavior. As a result, we relied on consumer recalls of food waste over the past month. While the study has some limitations, including relying on self-reported food waste behavior, its findings have important implications for future research. In terms of practice, the findings suggest the need for targeted interventions and policies to address food waste in the UAE. Efforts should be focused on younger generations and smaller households, as they are more likely to waste food. Social influencers, particularly men and elders could play a key role in changing behavioral patterns through public campaigns and community-based interventions. Furthermore, interventions should focus on addressing consumer attitudes towards food waste, including environmental concerns and personal attitudes. It is also possible to reduce household food waste by increasing consumer knowledge about food storage, preservation, and packaging, as well as improving food provisioning strategies to prevent over-purchasing. Further, investing in technology, such as smart apps that provide expiration dates and provide recipes based on ingredients available, can help individuals better manage their food and minimize waste. Future studies should examine the coherence between self-reported and observational measures of behavior. In order to change food waste habits and behaviors, future research is needed to assess the impact of key determinants, attitudes, behaviors, and emotions.

## 5. Conclusions

Food waste knowledge, attitudes, and behavior were examined in a first nationwide study in the United Arab Emirates. Whilst we found that positive consumer attitude towards avoiding FW are positively associated with behavioral intention (behavior attitude), this was not a good predictor of FW behavior in our study. Practice may be better determined by other factors, including food management skills, such as better meal planning and shopping to reduce over purchasing, having a greater awareness or knowledge of food safety, particularly safe handling and storage of leftover foods to help avoid food poisoning, practical constraints such as limited storage, and strong cultural or religious values. FW also appears to be more prevalent amongst younger generations (adults aged 18-30) and houses of multiple occupancy. More research involving a larger, more representative sample is, however, recommended before targeting such groups. Instead, government and policy makers should urgently consider developing public health campaigns, to educate people on safe storage of foods and product shelf-life to significantly reduce household consumer food waste in the UAE. Food manufacturers might be involved in such initiatives or be encouraged to provide better labelling. In parallel, incentives to encourage food manufacturers, retailers and consumers to avoid FW caused by over-purchasing and excess food preparation are needed, since individuals alone are not responsible for the problem of FW. In addition to reducing the economic, ethical, and sustainability issues associated with food waste, and ensuring future food security, urgent action will be required if the UAE is to contribute to achieving global sustainability.

## Data Availability

Figshare: The Attitudes and Practices of United Arab Emirates Consumers Towards Food Waste - A Nationwide Cross-Sectional Study. 10.6084/m9.figshare.23370104.v3. ^66^ This project contains the following underlying data:
- Food waste _Data Set_ Variables.xlsx - Food waste _ Anonymized Data.xlsx This project contains the following extended data:
- Extended data _Supplementary_Food waste.docx (supplementary tables and figures) Data are available under the terms of the Creative Commons Attribution 4.0 International license (CC-BY 4.0).

## References

[1] Bolwig S Tanner AN Riemann P : Reducing consumer food waste using green and digital technologies. 2021. Reference Source

[2] World Food Programme: 5 facts about food waste and hunger. 2020 [cited 2022 Sep 24]. Reference Source

[3] FAO: Food wastage footprint: impacts on natural resources- Summary Report. 2013. Reference Source

[4] Food FRSFR: *Losses and Waste in the Near East & North Africa Region.* FAO Cairo Egypt;2015.

[5] Tonini D Albizzati PF Astrup TF : Environmental impacts of food waste: Learnings and challenges from a case study on UK. *Waste Manag.* 2018 Jun 1;76:744–766. 10.1016/j.wasman.2018.03.032 29606533

[6] Campoy-Muñoz P Cardenete MA Delgado MC : Economic impact assessment of food waste reduction on European countries through social accounting matrices. *Resour. Conserv. Recycl.* 2017 Jul 1;122:202–209. 10.1016/j.resconrec.2017.02.010 Reference Source

[7] El Bilali H Ben Hassen T : Food Waste in the Countries of the Gulf Cooperation Council: A Systematic Review. *Foods.* 2020;9(4). 10.3390/foods9040463 32276529 PMC7230834

[8] The Paris Agreement|UNFCCC:[cited 2023 Feb 8]. Reference Source

[9] Abdelradi F : Food waste behaviour at the household level: A conceptual framework. *Waste Manag.* 2018 Jan 1;71:485–493. 10.1016/j.wasman.2017.10.001 Reference Source 29037881

[10] Zhongming Z Wei L : UNEP Food Waste Index Report 2021. 2021.

[11] Osaili TM Obaid RS Alqutub R : Food Wastage Attitudes among the United Arab Emirates Population: The Role of Social Media. *Sustainability.* 2022;14(3). 10.3390/su14031870

[12] Zamri GB Azizal NKA Nakamura S : Delivery, impact and approach of household food waste reduction campaigns. *J. Clean. Prod.* 2020 Feb 10;246:118969. 10.1016/j.jclepro.2019.118969

[13] UAE Ministry of Climate Change and Environment:[cited 2023 Feb 8]. Reference Source

[14] Timmermans AJM Ambuko J Belik W : Food losses and waste in the context of sustainable food systems. A report by the High Level Panel of Experts on Food Security and Nutrition of the Committee on World Food Security. Rome. 2014; pp.1–116. Report No.: 8. Reference Source

[15] Emirates Foundation:[cited 2023 Feb 8]. Reference Source

[16] Salem SB Preman JH : Food Supply Chain in Pandemic, Geopolitical, and Climate Change Era: Efforts of United Arab Emirates (UAE). *Agrotechnology.* 2022 Sep 26;2022(9):1–5. 10.31220/agriRxiv.2022.00142

[17] UAE’s Food Waste Pledge and Winnow Tackle Food Waste. *Food Tank.* 2020 [cited 2021 Sep 27]. Reference Source

[18] Dubai Municipality: UAE Food Bank. 2021 [cited 2022 Dec 15]. Reference Source

[19] Porpino G Parente J Wansink B : Food waste paradox: antecedents of food disposal in low income households. *Int. J. Consum. Stud.* 2015 Nov 1;39(6):619–629. 10.1111/ijcs.12207

[20] Pearson D Perera A : Reducing Food Waste: A Practitioner Guide Identifying Requirements for an Integrated Social Marketing Communication Campaign. *Soc. Mark. Q.* 2018 Mar 1;24(1):45–57. 10.1177/1524500417750830

[21] Elmenofi AGG Capone R Waked S : An exploratory survey on household food waste in Egypt. *Book of Proceedings of the VI international scientific agriculture symposium “Agrosym.* 2015; pp.15–18.

[22] Parfitt J Barthel M Macnaughton S : Food waste within food supply chains: quantification and potential for change to 2050. *Philos. Trans. R. Soc. B. Biol. Sci.* 2010 Sep 27 [cited 2023 Feb 8];365(1554):3065–3081. 10.1098/rstb.2010.0126 20713403 PMC2935112

[23] Abiad MG Meho LI : Food loss and food waste research in the Arab world: A systematic review. *Food Secur.* 2018;10(2):311–322. 10.1007/s12571-018-0782-7

[24] Economic SC for, Cooperation (COMCEC) CC of the O of I: *Reducing Food Waste in the OIC Countries.* Turkey: COMCEC Ankara;2017.

[25] Mattar L Abiad MG Chalak A : Attitudes and behaviors shaping household food waste generation: Lessons from Lebanon. *J. Clean. Prod.* 2018 Oct 10;198:1219–1223. 10.1016/j.jclepro.2018.07.085 Reference Source

[26] Ajzen I : The theory of planned behavior. *Theor. Cogn. Self-Regul.* 1991 Dec 1;50(2):179–211. 10.1016/0749-5978(91)90020-T Reference Source

[27] Fami HS Aramyan LH Sijtsema SJ : Determinants of household food waste behavior in Tehran city: A structural model. *Resour. Conserv. Recycl.* 2019;143:154–166. 10.1016/j.resconrec.2018.12.033

[28] Karim Ghani WAWA Rusli IF Biak DRA : An application of the theory of planned behaviour to study the influencing factors of participation in source separation of food waste. *Waste Manag.* 2013 May 1;33(5):1276–1281. 10.1016/j.wasman.2012.09.019 Reference Source 23415709

[29] Chalak A Abiad MG Diab M : The Determinants of Household Food Waste Generation and its Associated Caloric and Nutrient Losses: The Case of Lebanon. *PLoS One.* 2019 Dec 3;14(12):e0225789. 10.1371/journal.pone.0225789 31794574 PMC6890253

[30] Aktas E Sahin H Topaloglu Z : A consumer behavioural approach to food waste. *J. Enterp. Inf. Manag.* 2018 Jan 1;31(5):658–673. 10.1108/JEIM-03-2018-0051

[31] Janssen AM Nijenhuis-de Vries MA Boer EPJ : Fresh, frozen, or ambient food equivalents and their impact on food waste generation in Dutch households. *Waste Manag.* 2017 Sep;67:298–307. 10.1016/j.wasman.2017.05.010 Reference Source 28511926

[32] McCarthy B Liu HB : Food Waste and the ‘Green’ Consumer. *Australas. Mark. J.* 2017 May 1 [cited 2023 Feb 8];25(2):126–132. 10.1016/j.ausmj.2017.04.007

[33] Ghaziani S Ghodsi D Schweikert K : The Need for Consumer-Focused Household Food Waste Reduction Policies Using Dietary Patterns and Socioeconomic Status as Predictors: A Study on Wheat Bread Waste in Shiraz, Iran. *Foods.* 2022;11(18). 10.3390/foods11182886 36141014 PMC9498080

[34] Diaz-Ruiz R Costa-Font M Gil JM : Moving ahead from food-related behaviours: an alternative approach to understand household food waste generation. *J. Clean. Prod.* 2018 Jan 20;172:1140–1151. 10.1016/j.jclepro.2017.10.148 Reference Source

[35] Elshaer I Sobaih AEE Alyahya M : The Impact of Religiosity and Food Consumption Culture on Food Waste Intention in Saudi Arabia. *Sustainability.* 2021;13(11). 10.3390/su13116473

[36] Chammas G Yehya NA : Lebanese meal management practices and cultural constructions of food waste. *Appetite.* 2020 Dec 1;155:104803. 10.1016/j.appet.2020.104803 Reference Source 32791080

[37] Heidari A Mirzaii F Rahnama M : A theoretical framework for explaining the determinants of food waste reduction in residential households: a case study of Mashhad, Iran. *Env. Sci. Pollut. Res. Int.* 2020 Mar;27(7):6774–84. 10.1007/s11356-019-06518-8 31853853

[38] Attiq S Chau KY Bashir S : Sustainability of Household Food Waste Reduction: A Fresh Insight on Youth’s Emotional and Cognitive Behaviors. *Int. J. Environ. Res. Public Health.* 2021;18(13). 10.3390/ijerph18137013 34209149 PMC8293733

[39] Zahara KA Sari HH Setiawati TS : Knowledge, Attitudes, and Practice of Housewives As Members of the Environmental Community Against Household Food Waste Management. *E3S Web Conf.* 2021 [cited 2023 Feb 8];317:01066. 10.1051/e3sconf/202131701066 Reference Source

[40] Khorakian A Baregheh A Jahangir M : Household food waste prevention behavior: the role of religious orientations, emotional intelligence, and spiritual well-being. *J. Environ. Plan. Manag.* 2022 Aug 1;1–26. 10.1080/09640568.2022.2097062

[41] Karanja A Ickowitz A Stadlmayr B : Understanding drivers of food choice in low- and middle-income countries: A systematic mapping study. *Glob. Food Secur.* 2022 Mar 1;32:100615. 10.1016/j.gfs.2022.100615

[42] Stancu V Haugaard P Lähteenmäki L : Determinants of consumer food waste behaviour: Two routes to food waste. *Appetite.* 2016 Jan 1;96:7–17. 10.1016/j.appet.2015.08.025 26299713

[43] Baig MB Al-Zahrani KH Schneider F : Food waste posing a serious threat to sustainability in the Kingdom of Saudi Arabia–A systematic review. *Saudi J. Biol. Sci.* 2019;26(7):1743–1752. 10.1016/j.sjbs.2018.06.004 31762653 PMC6864160

[44] Aleshaiwi A Harries T . A step in the journey to food waste: How and why mealtime surpluses become unwanted. Appetite 2021 Mar;158:105040. 10.1016/j.appet.2020.105040 33188875

[45] Raosoft I: Sample size calculator by Raosoft, Inc. 2020.

[46] Association WM: World Medical Association Declaration of Helsinki: Ethical Principles for Medical Research Involving Human Subjects. *JAMA.* 2013 Nov;310(20):2191–2194. 10.1001/jama.2013.281053 24141714

[47] Richter B : Knowledge and perception of food waste among German consumers. *J. Clean. Prod.* 2017 Nov 10;166:641–648. 10.1016/j.jclepro.2017.08.009

[48] Visschers VHM Wickli N Siegrist M : Sorting out food waste behaviour: A survey on the motivators and barriers of self-reported amounts of food waste in households. *J. Environ. Psychol.* 2016 Mar 1;45:66–78. 10.1016/j.jenvp.2015.11.007

[49] Schanes K Dobernig K Gözet B : Food waste matters - A systematic review of household food waste practices and their policy implications. *J. Clean. Prod.* 2018 May 1;182:978–991. 10.1016/j.jclepro.2018.02.030

[50] Phasha L Molelekwa GF Mokgobu MI : Influence of cultural practices on food waste in South Africa—a review. *J. Ethn. Foods.* 2020 Oct 1;7(1):37. 10.1186/s42779-020-00066-0

[51] Abeliotis K Lasaridi K Chroni C : Attitudes and behaviour of Greek households regarding food waste prevention. *Waste Manag. Res.* 2014 Mar 1;32(3):237–240. 10.1177/0734242X14521681 24525671

[52] Beaton DE Bombardier C Guillemin F : Guidelines for the Process of Cross-Cultural Adaptation of Self-Report Measures. *Spine.* 2000;25(24):3186–3191. 10.1097/00007632-200012150-00014 Reference Source 11124735

[53] SPSS I: *IBM SPSS statistics for windows, version 28.0.* Armonk: IBM Corp;2021.

[54] Nunkoo R Bhadain M Baboo S : Household food waste: attitudes, barriers and motivations. *Br. Food J.* 2021 Jan 1;123(6):2016–2035. 10.1108/BFJ-03-2020-0195

[55] Wakefield A Axon S : “I’m a bit of a waster”: Identifying the enablers of, and barriers to, sustainable food waste practices. *J. Clean. Prod.* 2020 Dec 1;275:122803. 10.1016/j.jclepro.2020.122803

[56] UAE Food Bank- Facts and Figures: Dubai Municipality. [cited 2022 May 23]. Reference Source

[57] Flanagan A Priyadarshini A : A study of consumer behaviour towards food-waste in Ireland: Attitudes, quantities and global warming potentials. *J. Environ. Manag.* 2021 Apr 15;284:112046. 10.1016/j.jenvman.2021.112046 33540199

[58] Hebrok M Boks C : Household food waste: Drivers and potential intervention points for design – An extensive review. *J. Clean. Prod.* 2017 May 10;151:380–392. 10.1016/j.jclepro.2017.03.069

[59] Principato L Mattia G Di Leo A : The household wasteful behaviour framework: A systematic review of consumer food waste. *Ind. Mark. Manag.* 2021 Feb 1;93:641–649. 10.1016/j.indmarman.2020.07.010

[60] Pinto RS Pinto RM d S FFS M : A simple awareness campaign to promote food waste reduction in a University canteen. *Waste Manag.* 2018 Jun 1;76:28–38. 10.1016/j.wasman.2018.02.044 Reference Source 29503053

[61] Soma T Li B Maclaren V : Food Waste Reduction: A Test of Three Consumer Awareness Interventions. *Sustainability.* 2020;12(3). 10.3390/su12030907

[62] Aschemann-Witzel J De Hooge I Amani P : Consumer-Related Food Waste: Causes and Potential for Action. *Sustainability.* 2015;7(6):6457–6477. 10.3390/su7066457

[63] Groves RM Fowler FJ Jr Couper MP : *Survey methodology.* John Wiley & Sons;2011.

[64] Taherdoost H : Sampling methods in research methodology; how to choose a sampling technique for research. *Choose Sampl. Tech. Res. April.* 2016;10:2016. 10.2139/ssrn.3205035

[65] Schoeller DA Bandini LG Dietz WH : Inaccuracies in self-reported intake identified by comparison with the doubly labelled water method. *Can. J. Physiol. Pharmacol.* 1990 Jul 1;68(7):941–949. 10.1139/y90-143 2200586

[66] Kennedy L Khan MAB : The Attitudes and Practices of United Arab Emirates Consumers Towards Food Waste - A Nationwide Cross-Sectional Study.Dataset. *figshare.* 2023. 10.6084/m9.figshare.23370104.v3 PMC10905116 38434632

