# Supplementary material for: The attitudes and practices of United Arab Emirates consumers towards food waste: A nationwide cross-sectional study
Source: F1000Res. 2024 Feb 13;12:911. Originally published 2023 Jul 31. [Version 2] doi: 10.12688/f1000research.135678.2 (PMC10905116; doi:10.12688/f1000research.135678.2)
Supplement: Peer review history

# Reviewer report: Reviewer response for version 1

**DOI:** 10.5256/f1000research.148809.r225474

**Published:** 2024-02-08

**Authors:** Breda McCarthy

**Affiliations:** 1 Department of Economics and Marketing, James Cook University, Townsville City, Queensland, Australia

This article presents research on consumers' attitudes and practices in relation to food waste in the United Arab Emirates. Since studies of food waste in this geographic location are limited, the study is important from a consumer, policy and industry perspective, as well as from a sustainability perspective.

 Minor changes are recommended.

 The literature review is reasonable and the writing style is good.

 It is recommended that the authors remove the words on page 3, that "food loss and food waste are used interchangeably in the literature" since this is not exactly correct. Further, the definition of household food waste, in several quality publications, does not include inedible items, so I suggest the authors clarify what definition of food waste they use for their study, and the definition of food waste given to respondents in the survey. Refer to the distinction made between avoidable and unavoidable food waste (Secondi *et al.*, 2015 ^1^; https://wrap.org.uk/resources/report/household-food-and-drink-waste-united-kingdom-2021-22, Parfitt *et al.*, 2010). Most respondents would not see inedible food (egg shells, peelings) as food waste. Please reconsider the definition of food waste by referring to highly cited scholars in this field. It is also recommended that the scales used for the survey questions be given in the supplementary data section.

Is the work clearly and accurately presented and does it cite the current literature?

Yes

If applicable, is the statistical analysis and its interpretation appropriate?

Yes

Are all the source data underlying the results available to ensure full reproducibility?

Yes

Is the study design appropriate and is the work technically sound?

Yes

Are the conclusions drawn adequately supported by the results?

Yes

Are sufficient details of methods and analysis provided to allow replication by others?

Yes

Reviewer Expertise:

Consumer behaviour, food waste.

I confirm that I have read this submission and believe that I have an appropriate level of expertise to confirm that it is of an acceptable scientific standard.

## References

[1] : Household food waste behaviour in EU-27 countries: A multilevel analysis. *Food Policy* .2015;56: 10.1016/j.foodpol.2015.07.007 25-40 10.1016/j.foodpol.2015.07.007

# Reviewer report: Reviewer response for version 1

**DOI:** 10.5256/f1000research.148809.r225482

**Published:** 2023-12-14

**Authors:** Dalia Bedewy

**Affiliations:** 1 Ajman University, Ajman, Ajman, United Arab Emirates

This study seeks to understand the association between consumer attitudes, knowledge, and awareness relating to food waste practice of residents living in the UAE. to help inform policy and action for addressing this national priority.

 The aim of the study is clear, and the topic is demandingly important.

 The literature covers the socio-economic factors in a good way, however, a more detailed reflection to the psychological factors is neglected. Certain factors like compulsive buying attitude or other psychological factors could be included.

 The methodology and results are well presented; however, the conclusion needs to be more coherent.

Is the work clearly and accurately presented and does it cite the current literature?

Partly

If applicable, is the statistical analysis and its interpretation appropriate?

Yes

Are all the source data underlying the results available to ensure full reproducibility?

Yes

Is the study design appropriate and is the work technically sound?

Yes

Are the conclusions drawn adequately supported by the results?

Partly

Are sufficient details of methods and analysis provided to allow replication by others?

Yes

Reviewer Expertise:

educational psychology, social psychology

I confirm that I have read this submission and believe that I have an appropriate level of expertise to confirm that it is of an acceptable scientific standard.
